# A Programmable Soft Electrothermal Actuator Based on a Functionally Graded Structure for Multiple Deformations

**DOI:** 10.3390/polym17172288

**Published:** 2025-08-24

**Authors:** Fan Bu, Feng Zhu, Zhengyan Zhang, Hanbin Xiao

**Affiliations:** 1 School of Transportation and Logistics Engineering, Wuhan University of Technology, Wuhan 430063, China; 2 School of Mechanical Engineering, Hebei University of Technology, Tianjin 300401, China

**Keywords:** programmable soft electrothermal actuator, multiple deformations, functionally graded structure

## Abstract

Soft electrothermal actuators have attracted increasing attention in soft robotics and wearable systems due to their simple structure, low driving voltage, and ease of integration. However, traditional designs based on homogeneous or layered composites often suffer from interfacial failure and limited deformation modes, restricting their long-term stability and actuation versatility. In this study, we present a programmable soft electrothermal actuator based on a functionally graded structure composed of polydimethylsiloxane (PDMS)/multiwalled carbon nanotube (MWCNTs) composite material and an embedded EGaIn conductive circuit. Rheological and mechanical characterization confirms the enhancement of viscosity, modulus, and tensile strength with increasing MWCNTs content, confirming that the gradient structure improves mechanical performance. The device shows excellent actuation performance (bending angle up to 117°), fast response (8 s), and durability (100 cycles). The actuator achieves L-shaped, U-shaped, and V-shaped bending deformations through circuit pattern design, demonstrating precise programmability and reconfigurability. This work provides a new strategy for realizing programmable, multimodal deformation in soft systems and offers promising applications in adaptive robotics, smart devices, and human–machine interfaces.

## 1. Introduction

Soft actuators have emerged as vital components in the advancement of soft robotics, wearable technologies, and biomedical engineering, owing to their intrinsic compliance, adaptability, and enhanced safety in human–machine interaction [1,2,3]. In contrast to conventional rigid actuators, soft actuators can undergo continuous, reversible deformations and seamlessly adapt to irregular, unstructured environments. These features make them particularly suitable for applications such as minimally invasive, bioinspired grippers [4,5,6], artificial muscles [7,8,9], and smart textiles [10,11,12]. Among the various actuation mechanisms explored, electrothermal actuation has garnered growing interest [13,14,15,16]. This mechanism provides an effective pathway to achieving controlled deformation in soft systems while maintaining mechanical robustness and fabrication scalability.

Existing soft electrothermal actuators have been widely applied in soft robotics, smart wearable devices, and bioinspired actuation systems due to their advantages such as simple structure, low driving voltage, controllable response, and ease of integration [17,18]. Wang et al. [19] present a thermally induced shape reconfigurable soft actuator that shows reversible actuations with vast shape differences under thermal stimulus. Cao et al. [20] designed and manufactured a soft PI/PDMS actuator based on the electrothermal effect. By integrating micro-heaters through EHD printing technology, they achieved efficient bending actions under low-voltage drive and verified its practical functionality in soft robots. However, most conventional electrothermal actuators adopt either homogeneous material structures or simple layered composite architectures, where distinct interfacial boundaries exist between different layers. During repeated bending or complex deformation processes, these interface areas are prone to stress concentration, which can induce delamination, peeling, or performance degradation, seriously affecting the stability and lifespan of the actuators.

Moreover, most current electrothermal actuators are limited to simple deformation modes, primarily unidirectional bending or linear contraction [21,22], which limits their functionality in tasks requiring complex or multimodal movements. Hao et al. [23] designed a helical soft actuator with thermal stimulus response performance which can act as a robotic arm and lift heavy objects. Zhao et al. [24] present a soft bimorph actuator that has electrical and visual dual channel signal feedback functions for real-time multiplex motion perception. Although these designs demonstrate effective actuation performance, their deformation behaviors remain largely fixed and lack reconfigurability.

Therefore, improving the deformation versatility, localized actuation controllability, and long-term mechanical reliability of the soft electrothermal actuators remains a challenge. One promising strategy to address these limitations lies in the adoption of functionally graded structures [25,26,27]. Unlike traditional homogeneous or discretely layered architectures, functionally graded structures allow for the spatial gradation of material properties within a single continuous structure, which is achieved by varying the concentration of material [28,29,30,31]. This gradient enables spatial variation in thermal response and mechanical behavior across the actuator body, allowing for spatially programmable and coordinated deformations [32,33,34,35]. By strategically designing the internal distribution of conductive and elastic materials, functionally graded structure-based electrothermal actuators can achieve multiple deformation modes (such as bending, twisting, and elongation) in a single device without requiring complex external controls or multiple actuators.

In this work, we propose a programmable soft electrothermal actuator based on a functionally graded polydimethylsiloxane (PDMS)/multiwalled carbon nanotubes (MWCNTs) composite structure. By introducing spatial gradients in the distribution of MWCNTs within the PDMS matrix, the actuator exhibits position-dependent thermal and mechanical responses under electrical stimulation. By designing the layout of the conductive circuit, deformation modes such as L-shaped, U-shaped, and V-shaped bending can be predefined through simulations, enabling programmable and predictable actuation behavior. The proposed design demonstrates a novel strategy for achieving multimodal, reconfigurable, and programmable actuation in soft systems, while maintaining the advantages of simple fabrication and low-voltage operation. The results presented in this study provide new insights into the integration of functional material gradients for soft actuator development and open new opportunities for applications in soft robotics, adaptive systems, and human–machine interfaces.

## 2. Materials and Methods

### 2.1. Materials

The original polydimethylsiloxane (PDMS, Sylgard 184, Dow Corning Company, Midland, MI, USA) and 10% curing agent (Sylgard 184, Dow Corning Company, Midland, MI, USA) was mixed to the original PDMS to obtain the PDMS prepolymer. The eutectic gallium–indium alloy (EGaIn, composed of 75.5% gallium and 24.5% indium with a melting point of 16 °C) was sourced from Dongguan Wochang Metal Co., Ltd. (Dongguan, China). The multi-walled carbon nanotubes (MWCNTs), supplied by Suzhou Tanfeng Graphene Technology Co., Ltd. (Suzhou, China), have inner diameters ranging from 3 to 5 nm, outer diameters between 8 and 15 nm, and lengths varying from 3 to 12 μm. All these reagents were used without further purification.

### 2.2. Characterization

The resistance of EGaIn was observed with a digital multimeter (Fluke 8846A). Voltage was applied by a DC power supply (HSP-3005, Henghuiyuan Electronics Co., Ltd., Guangdong, China) and infrared thermography was performed using an infrared camera (H21pro, Beijing Pride Technology Co., Ltd., Beijing, China). A scanning electron microscope (SEM, ZEISS Smartzoom5, German Zeiss Group, Oberkochen, Germany) was used to observe the sample. MWCNT dispersion was prepared by an ultrasonic material disperser (SM-900C, Nanjing Shunma Instrument Co., Ltd., Naijing, China) Spin coaters (KW-4B, Beijing Saidkes Electronics Co., Ltd., Beijing, China) were used to prepare thin films.

### 2.3. Preparation of MWCNTs Dispersion

A total of 0.07 g of MWCNTs was placed in 20 mL CH_2_Cl_2_, and ultrasonic dispersion was carried out at 50% power (open 1 s off 2 s). The circulating water bath was set to 10 °C in the ultrasonic process, and the homogeneous dispersion of MWCNTs was obtained after 1 h of ultrasonic treatment. CH_2_Cl_2_ was selected because of its volatility, moderate polarity, and ability to wet and disperse MWCNTs effectively.

### 2.4. Preparation of PDMS/MWCNTs Composite Materials

The PDMS/MWCNT composite materials were prepared through a controlled solution-based mixing method. Specifically, an MWCNT dispersion with different mass fractions of MWCNTs was mixed with a predetermined mass of original PDMS, and the mixture was subjected to mechanical stirring at 500 rad/min for 4 h which facilitated complete evaporation of the solvent. After the solvent had completely evaporated, a curing agent was added to obtain PDMS/MWCNT composite materials.

### 2.5. Rheological Testing of PDMS/MWCNTs Composite Materials

The rheological properties of the PDMS/MWCNT composite materials were measured using a rotational rheometer with parallel plates of 20 mm in diameter with a clearance distance of 0.5 mm from the base plate. Before each test, the samples were heated up to 120 °C to eliminate historical temperatures and were then held at the test temperature for 5 min. During the oscillatory tests, the shear stress was swept from 10^−4^ Pa to 10 Pa at a fixed oscillation frequency of 1 Hz

### 2.6. Stress–Strain Tests of PDMS/MWCNTs Composite Materials

The stress–strain curves were measured using a universal testing machine equipped with a 200 N load cell, with a strain rate of 50 mm min^−1^. PDMS/MWCNTs film (gauge length 20 mm, width 5 mm, and thickness 1 mm) were tested at room temperature, and each measurement was repeated at least three times for reproducibility.

## 3. Results and Discussion

### 3.1. Design and Fabrication Process of the Electrothermal Actuator

**Figure 1:**
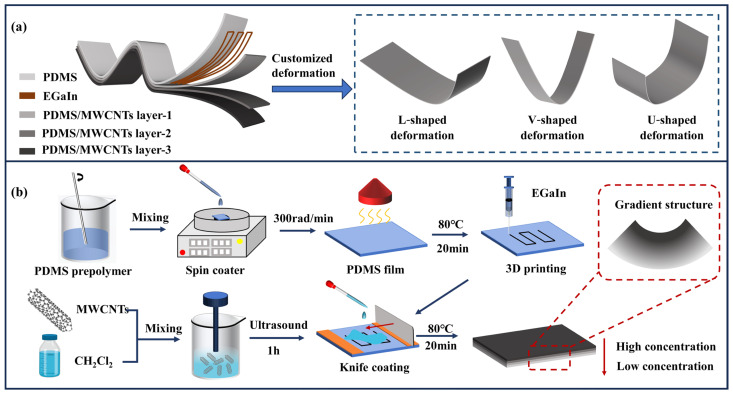
The programmable soft electrothermal actuator based on a functionally graded structure: (**a**) the diagram of the electrothermal actuator; (**b**) the preparation process of the electrothermal actuator.

The programmable electrothermal actuator based on a functionally graded structure is illustrated in Figure 1a. This actuator employs a five-layer hierarchical design: a printed EGaIn conductive circuit layer sandwiched between four PDMS/MWCNTs composite layers with gradationally tuned MWCNTs (the MWCNT concentrations from top to bottom in Figure 1a are, respectively, 0 wt%, 1 wt%, 2 wt%, and 3 wt%). In this work, the MWCNT content is expressed in weight percent (wt%), defined as the mass of MWCNTs relative to the total mass of PDMS and MWCNTs. The EGaIn layer serves as an embedded flexible electrode, while the stepwise increase in MWCNT content across the PDMS layer creates a discrete layer (stepwise, low–high).

The functionally graded structure of the soft electrothermal actuator features a continuous gradient of MWCNT concentration ranging from 0 wt% at the top to 3 wt% at the bottom within the PDMS matrix. This design establishes a progressively varying conductive network and coefficient of thermal expansion, enabling controlled asymmetric thermal strain upon electrical heating. Compared to conventional multilayer designs, this design significantly enhances interfacial bonding strength and mechanical reliability. The smooth gradient distribution ensures uniform strain transfer while preventing stress concentration. Specifically, the embedded EGaIn conductive circuit enables precise control over localized Joule heating which allows the realization of diverse complex deformations, including L-shaped, V-shaped, and U-shaped deformations. More importantly, the innovative “graded material and programmable circuitry” design synergistically combines structural stability with actuation efficiency.

The soft electrothermal actuator was prepared using coating and 3D printing, as shown in Figure 1b. The PDMS prepolymer was spin-coated at 300 rad/min for 1 min using a spin coater, followed by thermal curing in an oven at 80 °C for 20 min to obtain PDMS film with a thickness of 275 ± 15 μm. The EGaIn ink was printed on the PDMS film through a micro-nozzle (22 G) at room temperature, forming a programmable conductive circuit. The detailed printing parameters, including pressure, speed, and the distance between nozzle and substrate, follow our previous work [36]. Subsequently, the PDMS/MWCNT composite material with different concentration was sequentially blade-coated onto the PDMS film, with each layer thermally cured at 80 °C for 20 min in an oven. Each coating layer had a thickness of 300 μm. This layer-by-layer fabrication process successfully produced a programmable soft electrothermal actuator with functionally graded structure (20 mm × 5 mm × 1.2 mm).

### 3.2. Characterization of PDMS/MWCNTs Composite Materials

**Figure 2:**
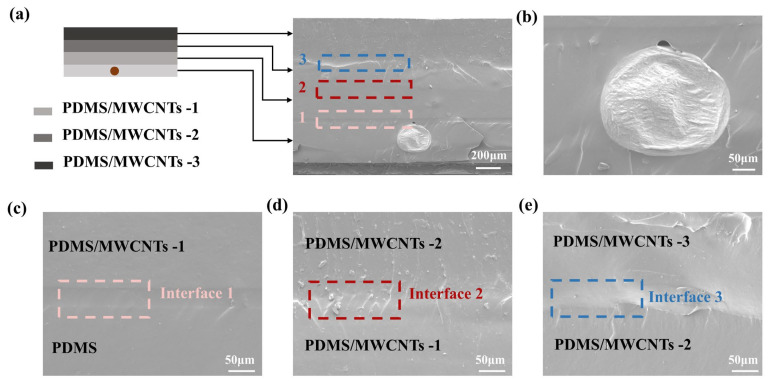
The SEM of the programmable soft electrothermal actuator: (**a**) cross-sectional view diagram of electrothermal actuator; (**b**) cross-sectional view of EGaIn conductive circuit; (**c**) the enlarged image of interface 1 between the PDMS layer and PDMS/MWCNTs-1 layer; (**d**) the enlarged image of interface 2 between the PDMS/MWCNTs-1 layer and the PDMS/MWCNTs-2 layer; (**e**) the enlarged image of interface 3 between the PDMS/MWCNTs-2 layer and the PDMS/MWCNTs-3 layer.

The cross-sectional view of the prepared electrothermal actuator is shown in Figure 2a, which adopts a multi-layer gradient structure composed of PDMS composite layers with varying MWCNT contents (labeled as PDMS, PDMS/MWCNTs-1, PDMS/MWCNTs-2, and PDMS/MWCNTs-3, respectively). SEM was employed to characterize the microscopic morphology of the EGaIn and material interfaces. As shown in Figure 2b, the EGaIn conductive circuit was completely embedded within the PDMS layer, and the cross-section was elliptical in shape. Figure 2c shows the interface 1 between the PDMS layer and the PDMS/MWCNTs-1 layer, which maintains excellent interfacial bonding. With increasing MWCNT content (Figure 2b,c,e), all interfaces shows continuous transitions without visible cracks or delamination, indicating strong interfacial bonding throughout the functionally graded structure.

**Figure 3:**
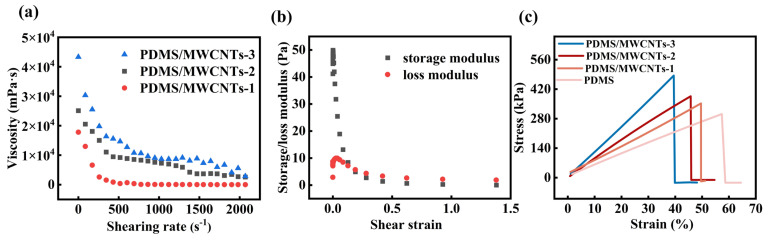
The rheological properties and mechanical properties of PDMS/MWCNT composite materials: (**a**) the viscosity curves of PDMS/MWCNT composite materials with different concentrations of MWCNTs; (**b**) the storage modulus and loss modulus of PDMS/MWCNT composite materials with a mass fraction of 1 wt%; (**c**) stress −strain curves of PDMS/MWCNT composite materials with different concentrations of MWCNTs.

The doping concentration of MWCNTs in the PDMS/MWCNT composite materials significantly influence its rheological. Figure 3a presents the shear-rate-dependent viscosity behavior of PDMS/MWCNT composites at three different MWCNT concentrations (1 wt%, 2 wt%, and 3 wt%). The results demonstrate that the viscosity of the PDMS/MWCNT composite material decreases with increasing shear rate, exhibiting a shear-thinning behavior characteristic of non-Newtonian fluids. Notably, higher MWCNT loading leads to greater initial viscosity, indicating that the filler network substantially enhances flow resistance. The storage modulus (G’) and loss modulus (G’’) of the PDMS composite material containing 1 wt% MWCNTs under varying shear strains is shown in Figure 3b. It can be observed that G’ consistently exceeds G’’ in the low-strain region, indicating that the PDMS/MWCNT composite material exhibits predominantly elastic, solid-like behavior. As the strain increases, both moduli gradually decrease, suggesting the breakdown of the filler network structure at high strains. This phenomenon further confirms that MWCNTs form an effective reinforcing network within the PDMS matrix, thereby significantly enhancing the mechanical stability of the PDMS/MWCNT composite material.

In addition to rheological properties, the content of MWCNTs in the PDMS/MWCNT composite materials also influences the mechanical performance. As demonstrated in Figure 3c, the tensile strength of PDMS/MWCNT composite materials shows a marked improvement with increasing MWCNTs content. This mechanical enhancement can be attributed to three primary mechanisms [37]: (1) the effective stress transfer at the CNT-PDMS interface, (2) the restriction of polymer chain mobility by the nanoscale fillers, and (3) the formation of a percolating CNT network that bears external loads. However, this strengthening effect is accompanied by a gradual reduction in elongation at break, revealing the characteristic strength–ductility trade-off common in nanoparticle-reinforced elastomers. The 3 wt% composite exhibits optimal mechanical properties, achieving a balance between enhanced stiffness and maintained deformability. These mechanical property modifications confirm that MWCNTs serve as multifunctional fillers that simultaneously alter both the processing characteristics (rheology) and end-use performance (mechanics) of the elastomeric PDMS/MWCNT composite material.

### 3.3. Performance of Programmable Soft Electrothermal Actuator

**Figure 4:**
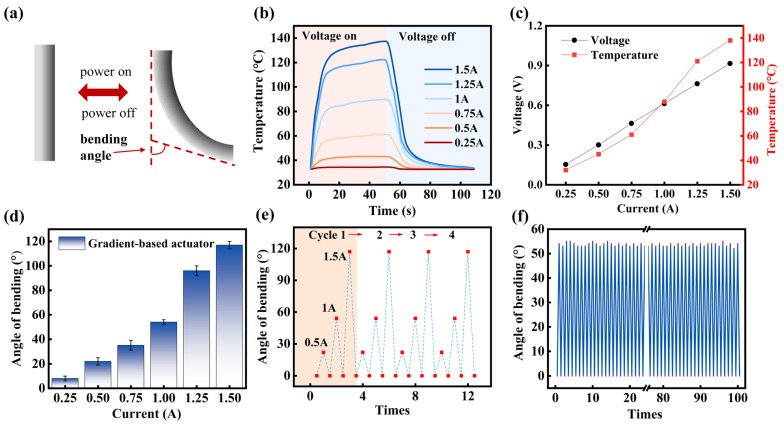
The performance of the programmable soft electrothermal actuator: (**a**) schematic illustration of the actuation mechanism under voltage stimuli; (**b**) temperature change curves of the electrothermal actuator under different currents; (**c**) curves of resistance and temperature changes under different currents; (**d**) the bending angle of the actuator under different currents; (**e**) changes in bending angle under different currents during four cycles; (**f**) multiple cycles of the electrothermal actuator under 1.0 A.

The programmable soft electrothermal actuator based on functionally graded structure bends is owing to the asymmetric thermal strain caused by gradient distribution of the material’s coefficient of thermal expansion (CTE). As shown in Figure 4a, when current passes through the EGaIn conductive circuit and generates Joule heating, the gradient distribution of MWCNT concentration across the layers (from PDMS to high-concentration PDMS/MWCNTs) creates a continuous CTE gradient (the pure PDMS layer has the highest CTE, while the high-concentration MWCNTs layer has the lowest). This CTE gradient causes differential thermal expansion strains under the same temperature rise: the PDMS layer exhibits the greatest expansion, the high-concentration MWCNTs layer the least, with intermediate layers showing continuous strain variation. Simultaneously, the gradient distribution of MWCNTs also leads to gradients in thermal conductivity and elastic modulus: the high-concentration MWCNT layer has higher thermal conductivity (faster heat dissipation) and greater elastic modulus (higher stiffness). This not only affects the temperature field distribution but also shifts the neutral axis toward the low-strain side, further amplifying the bending effect. Through strong interfacial bonding, strain energy is progressively transferred and accumulated between layers, ultimately forming a continuous strain gradient along the thickness direction. This generates a net bending moment directed toward the high-concentration MWCNT layer, resulting in controllable directional bending deformation of the actuator. Compared to traditional bilayer structures, this gradient design offers advantages such as smoother strain transition, more uniform stress distribution, and greater deformation magnitude. As shown in Figure 4a, the bending angle was measured by capturing images of the bent samples using a camera or microscope. ImageJ 2025 software was used to mark key points (fixed end, free end, and bending point) to calculate the resulting angle.

To investigate the response speed of the soft electrothermal actuator, temperature variations with time were measured at different current input (0.25 A, 0.5 A, 0.75 A, 1.0 A, 1.25 A, and 1.5 A). As shown in Figure 4b, the experimental results demonstrate that the temperature rises rapidly during the initial first 8 s, after which, the temperature dropped sharply within 50 s when the voltage turned off. Furthermore, increasing the current leads to significantly higher saturation temperatures while slightly prolonging the time required to reach thermal equilibrium. These findings confirm that the heating characteristics and response speed of the actuator can be effectively regulated through current adjustment. Figure 4c shows the changes in EGaIn conductive circuit resistance and temperature under different currents. It can be observed that the voltage and current of the EGaIn conductive circuit exhibit an approximately linear relationship, indicating that the resistance of the EGaIn conductive circuit maintains relatively stable characteristics.

The bending angle was also investigated to study the vending performance of the soft electrothermal actuator. In the experiment, different currents (0.25 A, 0.5 A, 0.75 A, 1.0 A, 1.25 A, and 1.5 A) were applied to EGaIn to explore the relationship between current and bending angle. As shown in Figure 4d, the bending angle is positively correlated with current, and when the current reaches 1.5 A, the bending angle increases to 117°. To investigate the reliability of the soft electrothermal actuator under cyclic working conditions, the vending performance under varying currents (0.5 A, 1.0 A, and 1.5 A) was studied in Figure 4e. The results demonstrate that during four testing cycles, the bending angle of the soft electrothermal actuator increased significantly with higher currents while maintaining stable values in each cycle, indicating excellent current responsiveness and cyclic stability. Subsequently, the long-term performance under a constant current of 1.0 A was studied. After 100 cycles test (Figure 4f), the soft electrothermal actuator maintained stable bending angles without noticeable performance degradation, confirming its reliability and durability under steady working conditions.

### 3.4. The Programmable Deformation of the Soft Electrothermal Actuator

The programmable deformation of the soft electrothermal actuator is achieved through a combination of Joule heating and asymmetric structural design. When the voltage is applied to the embedded EGaIn conductive circuit, resistive heating occurs, causing localized thermal expansion in the layers with gradient structure. This expansion is constrained by an adjacent passive layer, creating a bending moment that deforms the structure. By strategically patterning the conductive circuit and precisely controlling the current magnitude (0.5–1.5 A), different regions of the actuator can be selectively activated to produce complex deformance (L-shaped, U-shaped, and V-shaped).

**Figure 5:**
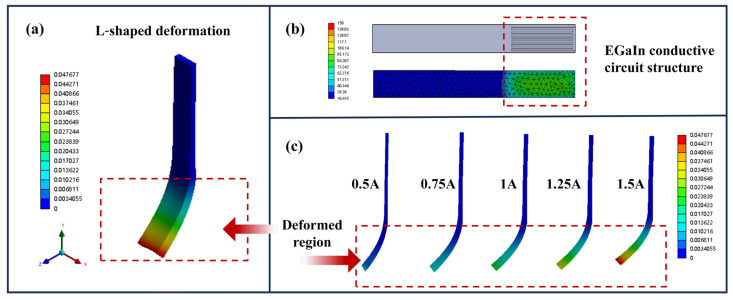
The L-shaped deformation of the programmable soft electrothermal actuator: (**a**) schematic diagram of the actuator deformation simulation; (**b**) the EGaIn conductive structure of the L-shaped deformation; (**c**) different L-shaped deformations under different current conditions.

To investigate the multiple deformation capabilities of soft electrothermal actuators, The ABAQUS (version 2025) finite element analysis was used to simulate the deformation behavior of actuators with different EGaIn conductive circuit structures. For the simulation parameters, we have followed the guidelines and settings detailed in our previous work [36]. These parameters ensured consistency in the simulation process and aligned with the methodology used in earlier studies. As shown in Figure 5a, the L-shaped deformation occurred when the EGaIn conductive circuit structure was designed at the end of the actuator (Figure 5b). Due to the local conductive structure design, the expanding active layer is mechanically constrained by the adjacent passive layer, resulting in an uneven strain distribution at the end that produces an L-shaped bending deformation toward the passive layer side. By adjusting the input current (0.5 A to 1.5 A), precise control over the deformation can be achieved.

**Figure 6:**
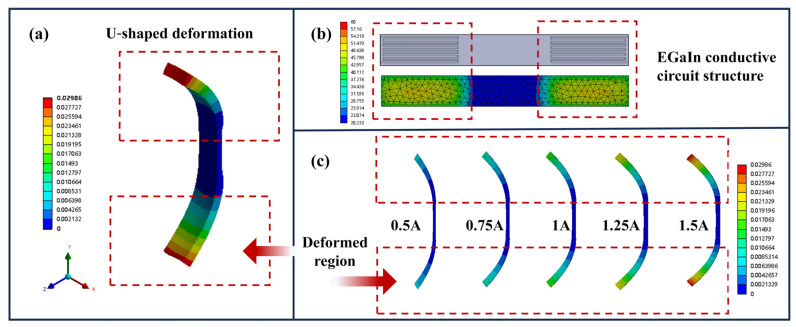
The U-shaped deformation of programmable soft electrothermal actuator: (**a**) schematic diagram of actuator deformation simulation; (**b**) the EGaIn conductive structure of the U-shaped deformation; (**c**) different U-shaped deformations under different current conditions.

Figure 6 illustrates the U-shaped deformation characteristics and working mechanism of the soft electrothermal actuator. When the EGaIn conductive circuit was printed on both ends of the actuator to ensure uniform current distribution (Figure 6b), Joule heating occurs simultaneously at both terminals upon electrification, creating asymmetric active expansion. Constrained by the central passive layer, the expanding regions at both ends bend uniformly in the same direction, ultimately forming a characteristic U-shaped deformation. This symmetric drive design achieves more stable U-shaped configuration through synchronized thermal expansion at both ends, with bending curvature increasing significantly as current rises (0.5 A–1.5 A) in Figure 6c. Compared to single-end actuation, the dual-end symmetric heating produces more uniform deformation, offering unique advantages for applications requiring symmetrical shape changes like soft grippers.

**Figure 7:**
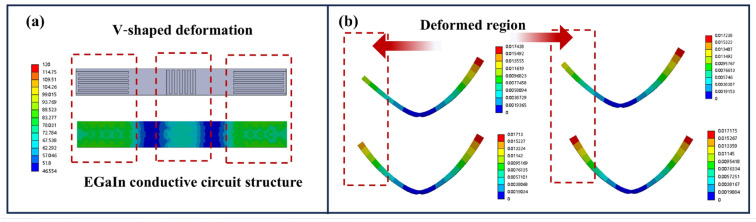
The V-shaped deformation of the programmable soft electrothermal actuator: (**a**) the EGaIn conductive structure of the U-shaped deformation; (**b**) different V-shaped deformations under different current conditions.

The V-shaped deformation of the programmable soft electrothermal actuator is shown in Figure 7. The specially designed EGaIn conductive circuit structure (Figure 7a) generates asymmetric thermal expansion through Joule heating when electrified. The schematic of the deformation zone shows that this structural design enables directional bending to form V-shaped configurations. As shown in Figure 7b, the actuator exhibits adjustable V-shaped deformation under 0.25 A to 1 A.

## 4. Conclusions

In this study, we developed a programmable soft electrothermal actuator based on a functionally graded PDMS/MWCNT composite structure embedded with an EGaIn conductive circuit. The gradient distribution of MWCNTs enabled spatially varying mechanical and thermal properties, which effectively enhanced interfacial bonding and mechanical stability compared to traditional layered designs. The actuator demonstrated excellent actuation performance, including rapid thermal response, stable cyclic durability, and programmable multimodal bending deformations (L-shaped, U-shaped, and V-shaped), achieved through circuit pattern design. Our results confirm that functionally graded structures provide a powerful strategy for achieving smooth strain gradients, uniform stress distribution, and complex programmable deformation modes in soft electrothermal actuators. This work not only advances the design and fabrication of robust, versatile soft actuators but also opens new avenues for their applications in adaptive robotics, wearable devices, and human–machine interfaces.

## Data Availability

The original contributions presented in this study are included in this article. Further inquiries can be directed to the corresponding authors.
